# Temporal trends in the burden of non-communicable diseases in countries with the highest malaria burden, 1990–2019: Evaluating the double burden of non-communicable and communicable diseases in epidemiological transition

**DOI:** 10.1186/s12992-022-00882-w

**Published:** 2022-10-23

**Authors:** Zhuo Li, Junyi Shi, Na Li, Minmin Wang, Yinzi Jin, Zhi-jie Zheng

**Affiliations:** 1 Department of Global Health, School of Public Health, Peking University, 38 Xue Yuan Road, Haidian District, 100191 Beijing, China; 2 Institute for Global Health and Development, Peking University, Beijing, China

**Keywords:** Malaria, Non-communicable disease, Double burden of disease

## Abstract

**Background:**

Non-communicable diseases (NCDs) are rapidly increasing in sub-Saharan African countries, where 96% of global malaria deaths occur. This study aimed to investigate the disease burden of NCDs in countries with the current highest malaria mortality.

**Methods:**

Data for this study were obtained from the Global Burden of Disease 2019 study (1990–2019). We selected the ten countries with malaria’s highest age-standardised mortality rate (ASMR) and identified and ranked the five NCDs with the highest ASMR in each country. Measures of the NCDs disease burden included ASMR, age-standardised disability-adjusted life-years (DALY), years of life lost (YLL) and years lost due to a disability (YLD). The Estimated annual percentage change (EAPC) was used to examine the trends of the NCDs disease burden from 1990 to 2019.

**Results:**

As of 2019, the ASMR of chronic liver disease, kidney disease, diabetes mellitus, Alzheimer’s disease and other dementias, hypertensive heart disease and stroke were higher than the global average. From 1990 to 2019, the ASMR for Alzheimer’s disease and other dementias, type II diabetes mellitus, and chronic kidney disease increased by 3.0%, 10.8%, 13.3%, and the age-standardised DALY rate increased by 3.7%, 27.6%, 6.3%, and the increases tended to be in younger populations.

**Conclusion:**

The double burden of non-communicable and communicable diseases is crippling the health systems of many sub-Saharan African countries and is often neglected. The prevention, surveillance, and control of diseases require an integrated strategy, with governments and non-government organisations aligned and supported by the global initiative.

**Supplementary Information:**

The online version contains supplementary material available at 10.1186/s12992-022-00882-w.

## Introduction

Malaria as one of the most serious communicable diseases and threatens more than half of the world’s population [1]. In 2020, there were an estimated 241 million fall ill from malaria, in comparison with 1.5 million people infected with human immunodeficiency virus, and 9.9 million contract tuberculosis [2–4]. According to World Health Organization’s (WHO) latest World Malaria Report, there were an estimated 627 000 malaria deaths worldwide in 2020, and 96% were respectively from countries in sub-Saharan Africa [5]. Despite significant progress in reducing the overall burden of malaria over the past decade, certain populations continue to experience higher disease mortality and morbidity rate and relatively low access to life-saving interventions. Moreover, non-communicable diseases (NCDs) are now emerging or rapidly increasing and mainly affect vulnerable groups, which carries a huge burden that impairs the economic and social development of the sub-Saharan Africa region [6]. According to the WHO, about 77% of NCDs deaths occur in low- and middle-income countries (LMICs) [7]. With the population ageing, rising prevalence of multiple-morbidity and longer life expectancy, an increasing number of people are expected to bear the health burden of NCDs [8]. By 2030, the number of deaths from NCDs in Africa is projected to exceed that from communicable diseases and perinatal deaths combined [9]. Many countries in sub-Saharan Africa are experiencing a double burden of non-communicable and communicable diseases, which poses significant health challenges for populations, weakens health systems, and severely hinders the United Nations Sustainable Development Goals [10].

Over the past few decades, the double burden of disease in LMICs and underfunding and investment in NCDs has been identified and documented as a recognized problem by the WHO [11, 12]. Communicable diseases such as malaria have captured much global attention and funding commitments [13]. Many national governments and international organisations are committed to cooperation among communicable disease prevention and control programs, while support and efforts in NCD care are relatively inadequate [14]. The prevention and control of NCDs should share equal importance with communicable diseases [15]. Prior studies have indicated that NCDs may be associated with malaria and malaria severity [16–18]. In several studies from Africa, malaria has been documented to be more common in people with diabetes [19]. In addition, few experimental studies have also shown that malaria may affect blood pressure and induce hypertension, which contributes to heart failure [20, 21]. However, there are few studies on the trends in the burden of NCDs in countries with the highest malaria burden from the perspective of epidemiological transition.

To fill this gap, this study aimed to investigate the temporal trends in the burden of NCDs in countries with the highest malaria burden between 1990 and 2019. The findings of our study can provide evidence that the sub-Saharan African countries are faced with a double burden of non-communicable and communicable diseases in epidemiological transition. We also report how the trends in the burden of different NCDs vary by country, age, and sex, so that countries could identify priority groups in policymaking for the prevention, surveillance, and management of non-communicable and communicable diseases, thereby facilitating the achievement of the United Nations Sustainable Development Goals.

## Methods

### Data sources

The data of this study was obtained in the Global Burden of Diseases (GBD) 2019 public datasets, which are available from http://ghdx.healthdata.org/gbd-results-tool (accessed on May 22, 2022). The GBD is research led by the Institute for Health Metrics and Evaluation (IHME) of the University of Washington, dedicated to measuring disability and death from various causes worldwide [22]. The GBD 2019 public dataset were based on secondary data from existing registriers [22]. Estimates of mortality were calculated from available data and models when reliable registry data were lacking or reporting lags occurred. We extracted the annual number of deaths and mortality rates of malaria of infectious diseases from 1990 to 2019, by sex, age, and geography. The ten countries with the highest age-standardized mortality rate (ASMR) for malaria were identified. All available data on causes of death were standardized and pooled into a single database. The five NCDs with the highest ASMR in each country were then identified and ranked, including ischemic heart disease, stroke, diabetes mellitus, chronic liver diseases, chronic kidney disease, Alzheimer’s disease and other dementias, chronic obstructive pulmonary disease, and hypertensive heart diseases.

### Statistical analysis

Measures of the NCDs disease burden included ASMR, age-standardized disability-adjusted life-years (DALY), years of life lost (YLL) and years lost due to a disability (YLD). The Estimated annual percentage change (EAPC) was used to examine the trends of the NCDs disease burden from 1990 to 2019.

EAPC is a method proposed by Hankey to measure trends in age-standardized rates (ASR) over time [23]. Joinpoint regression analysis (Joinpoint regression software, Version 4.8.0.1 –April 2020) [24] was performed in this study to calculate EAPC quantify disease mortality trends between 1990 and 2019. The calculation methods were: first, a regression line was fitted to the natural logarithm of the rates, i.e., y = α + βx + ε, where y = ln (ASMR) and x = calendar year; then, the EAPC was calculated as 100×(eβ − 1), with 95% confidence interval (CI) obtained from the linear regression model. For trend description, the term ‘increase’ was used when the EAPC estimation and the lower boundary of its 95% CI were both > 0. In contrast, the term ‘decrease’ was used when the EAPC estimation and the upper boundary of its 95% CI were both < 0. Otherwise, the term ‘stable’ was used. The IBM Statistical Package for social science (SPSS) version 26.0 was used for data analysis.

## Results

**Fig. 1:**
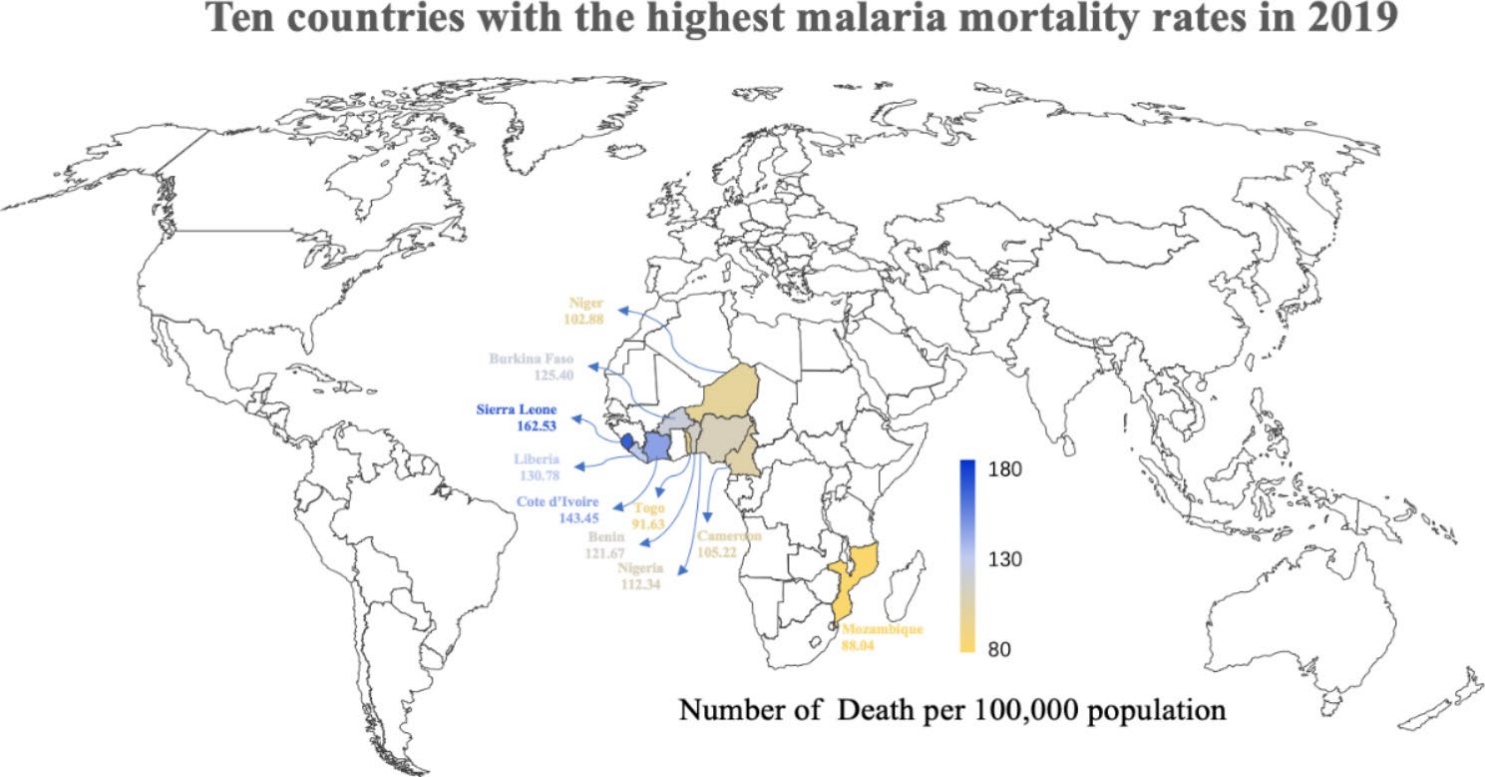
Top ten countries with the highest malaria mortality rates in 2019, which were Nigeria, Liberia, Burkina Faso, Niger, Cote d’Ivoire, Benin, Sierra Leone, Togo, Cameroon and Mozambique. It is worth noting that these countries with the highest burden of malaria deaths are all located in the African Region

Ten countries with the highest malaria mortality rates in 2019 were selected, which were Nigeria, Liberia, Burkina Faso, Niger, Cote d’Ivoire, Benin, Sierra Leone, Togo, Cameroon, Mozambique. It is worth noting that these countries with the highest burden of malaria deaths are all located in the African Region (Fig. 1).

**Fig. 2:**
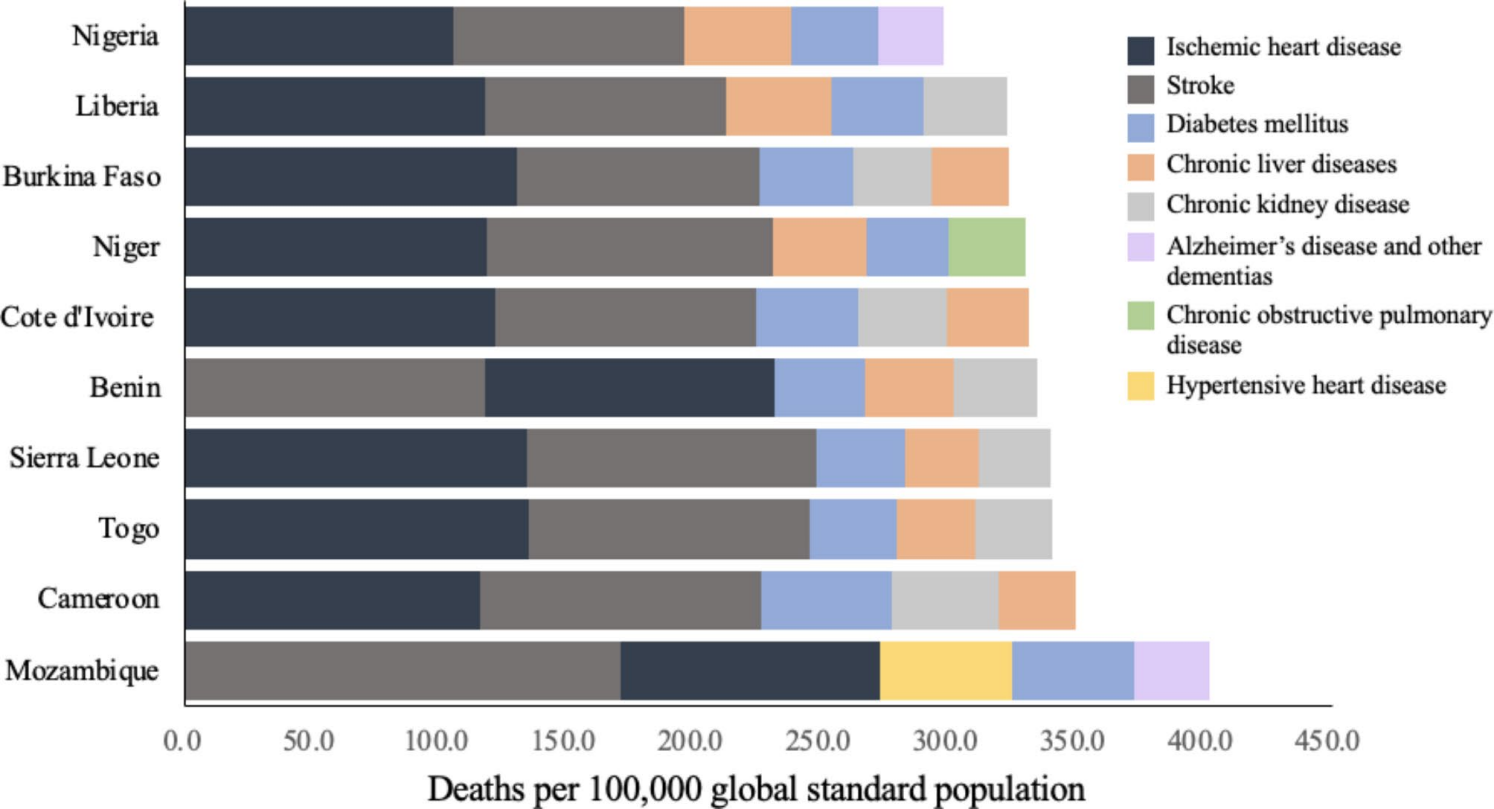
Age-standardized death rates for the eight leading NCDs in the ten selected countries, 2019. Including ischemic heart disease, stroke, diabetes mellitus, chronic liver diseases, chronic kidney disease, Alzheimer’s disease and other dementias, chronic obstructive pulmonary disease, and hypertensive heart disease. Of these eight diseases, ischemic heart disease, stroke and diabetes mellitus were the most common leading causes of death in all ten countries. The contribution (%) of the top five NCDs with the highest mortality rate to the total mortality rate of each selected country and the world is shown in Appendix 1

Among the ten selected countries, the top five NCDs with the highest ASMR in each country are shown in the Fig. 2, including ischemic heart disease, stroke, diabetes mellitus, chronic liver diseases, chronic kidney disease, Alzheimer’s disease and other dementias, chronic obstructive pulmonary disease, and hypertensive heart disease. Of these eight diseases, ischemic heart disease, stroke and diabetes mellitus were the most common leading causes of death in all ten countries. In 2019, the top five NCDs with the highest ASMR in each of the ten selected countries together account for 10% of the total mortality rate in their respective countries. The eight NCDs together accounted for 46.3% of global mortality (Male = 44.7%, Female = 48.4%). Females had a higher mortality rate across the eight NCDs globally, while males were at greater risk of death in the African region (Appendix Table 1).

During the period between 1990 and 2019, among the selected countries, the ASMR of chronic liver disease, kidney disease and diabetes mellitus (both type I and type II) were higher than the global average. Notably, ASMR for type I diabetes mellitus declined in all selected countries except Mozambique; ASMR for type II diabetes mellitus increased in all selected countries. This article therefore focuses on the growth trend of type II diabetes mellitus (Appendix Figs. 1 and 2**).**

**Fig. 3:**
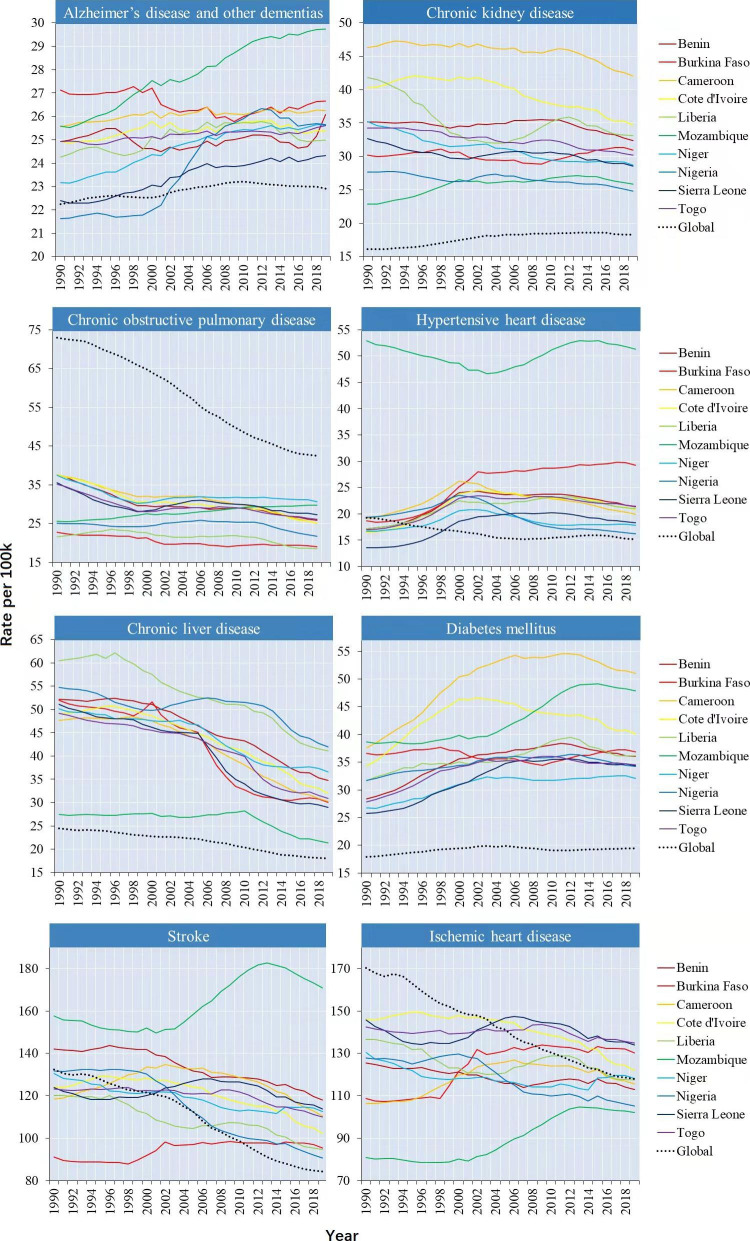
The long-term mortality trends (1990–2019) of the eight leading NCDs in the ten countries During 1990 and 2019, among the selected countries, the ASMR of chronic liver disease, kidney disease and diabetes mellitus were higher than the global average. As of 2019, in addition to the above three diseases, the ASMR of Alzheimer’s disease and other dementias, hypertensive heart disease and stroke also exceeded the global average From 1990 to 2019, the ASMR for Alzheimer’s disease and other dementias, diabetes mellitus, and chronic kidney disease increased by 3.0%, 8.6%, 13.3%

**Fig. 4:**
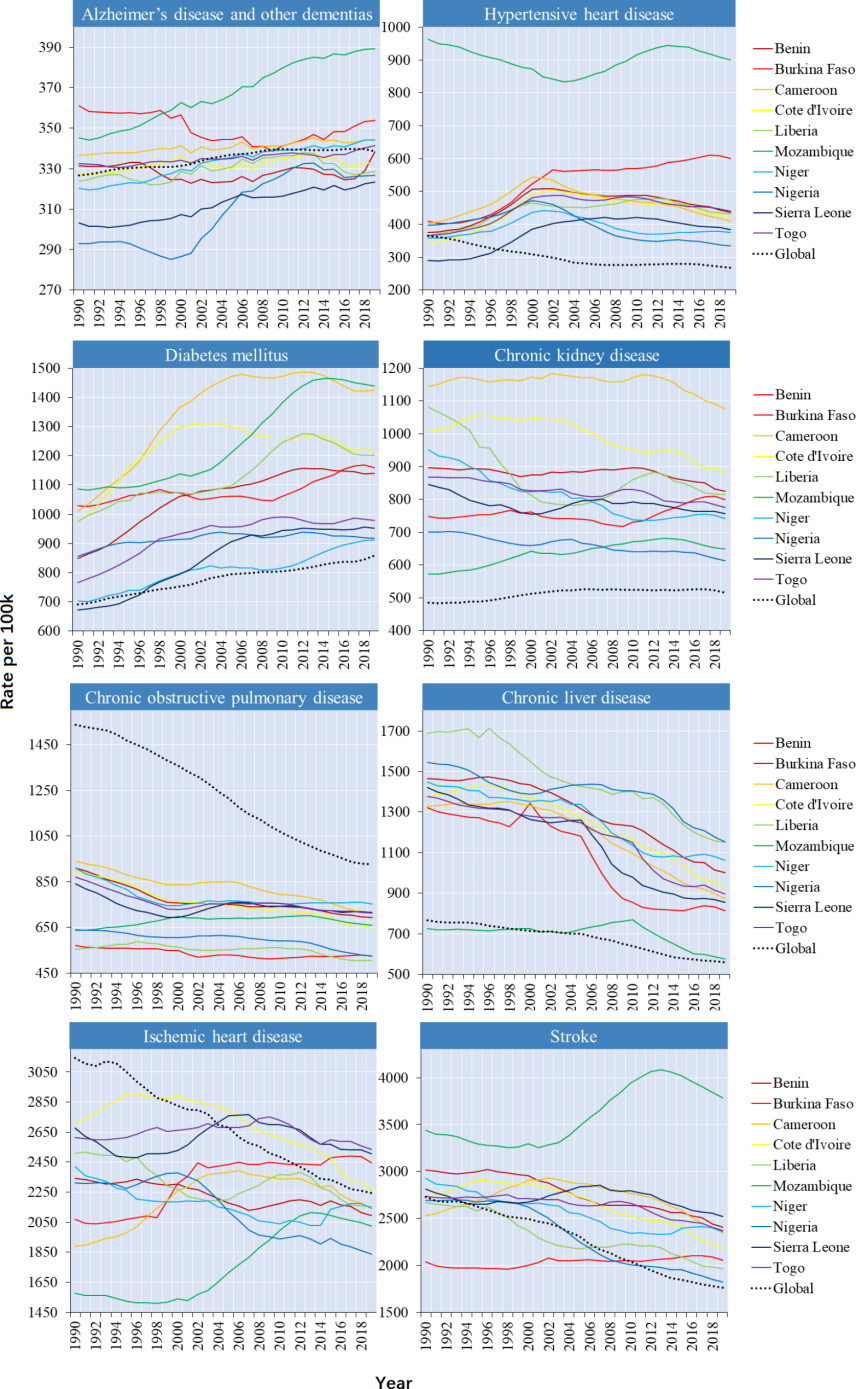
Trends of the age-standardized DALYs due to the eight leading NCDs in the ten countries, 1990–2019. From 1990 to 2019, the age-standardized DALY rate for Alzheimer’s disease and other dementias, diabetes mellitus, and chronic kidney disease increased by 3.7%, 24.4%, 6.3%

As of 2019, in addition to the above three diseases, the ASMR of Alzheimer’s disease and other dementias, hypertensive heart disease and stroke also exceeded the global average. During the period between 1990 and 2019, the global ASMR for Alzheimer’s disease and other dementias, diabetes mellitus, and chronic kidney disease increased by 3.0%, 8.6% (type II: 10.8%), 13.3%; the age-standardized DALY rate increased by 3.7%, 24.4% (type II: 27.6%), 6.3% **(**Figs. 3 and 4**)**. Globally, the increase in mortality from these three diseases was mainly in the middle-aged and elderly population. However, ASMR increases in Alzheimer’s disease, type II diabetes mellitus, and chronic kidney disease tended to be in younger populations in most of the selected countries).

For Alzheimer’s disease and other dementias, Nigeria had the largest increases in ASMR at 18.7% (EAPC = 0.8468, 95% CI: 0.7110 ~ 0.9828) followed by Mozambique at 16.2% (EAPC = 0.5788, 95% CI: 0.5444 ~ 0.6131); The largest increase was between the age of 44–69 in both countries. Mozambique had the largest increases of Alzheimer’s disease and other dementias in age-standardize DALYs rate (12.8%). The age-standardized DALYs rate increased most significantly in diabetes mellitus at 24.4% (type II: 27.6%). The ASMR of type II diabetes mellitus increased with age after age 35, and the highest increase was between the age of 85–89. Among the ten countries, Cameroon had the most significant increase in ASMR of type II diabetes mellitus at 38.9% (EAPC = 1.2072, 95% CI: 0.8529 ~ 1.4997), followed by Sierra Leone at 36.5% (EAPC = 1.2720, 95% CI: 1.0050 ~ 1.5113). The global ASMR of chronic kidney disease increased higher in women than in men. Cameroon had the highest ASMR of chronic kidney disease at 42.0 per 100,000 population. Mozambique had the most significant increase in ASMR at 13.3% (EAPC = 0.4963, 95% CI: 0.3602 ~ 0.6327) followed by Burkina Faso at 2.6% (EAPC = 0.0321, 95% CI: -0.0738 ~ 0.1381), and the increase tended to be in younger populations in these two countries (Appendix Figs. 3 and 4; Appendix Table 2).

## Discussions

The ten countries with the highest malaria burden in 2019 were all from LMICs in the African region. Even though malaria is a preventable and treatable communicable disease, there are challenges to control on the long road to elimination [3, 25], it continues to have a devastating impact on the health and livelihoods of people around the world, driven by multiple factors, including local weather, the economy, and the fragile health system in these malaria-endemic countries [26, 27]. Over the past few decades, communicable diseases such as malaria have captured much of the global attention and resources [28]. Numerous bilateral and multilateral cooperation has been carried out to expand the scope of malaria prevention and control and reduce the burden of malaria on people’s lives [29]. In contrast to communnicable diseases, NCDs in these malaria-endemic countries are often neglected [30], even though these diseases together account for 10% of total national mortality. The statistics of WHO in 2020 show that compared with the prevention and control of communicable diseases, the progress of NCDs prevention and control is relatively lagging behind [31]. Moreover, with the rapid spread of COVID-19 around the world, 75% of countries reported that NCDs services were disrupted to varying degrees, and the capacity of health systems to prepare for and respond to these diseases has been threatened, especially for vulnerable populations who require regular and long-term healthcare [32]. Mortality rates from NCDs are likely to rise further due to disruptions in healthcare services during the pandemic [33]. Hence, the double disease burden needs urgent attention from all sectors of society.

Prior studies have indicated that some types of NCDs may be associated with malaria and malaria severity [16–18]. A Danish study found that malaria may be associated with cardiac complications, and people with malaria history may carry out a long-term risk of cardiovascular conditions and deaths [21]. Two recent studies also have suggested that long-term malaria exposure is linked to cardiovascular events, especially hypertension [17, 34] Moreover, Kalra et al. suggested that malaria has been more common in diabetes mellitus [19]. Malarial infection during pregnancy is a critical cause of low birth weight and anaemia. Placental malaria and anaemia may induce hypoxia and thereby negatively affect foetal growth. This could be a potential cause of type II diabetes mellitus later in life [35]. Currently, there is limited research on the relationship between malaria and alzheimer’s disease and other dementias, chronic kidney and liver disease in areas with high malaria burden. Future studies are warranted to explore the links between malaria and these NCDs. Additionally, urgent attention needs to be paid to the structural factors- politics, economy, demographic situation, resource allocation for health care behind the convergence of these NCDs and malaria [36, 37].

Malaria and NCDs require adherence to both prevention and treatment regimes. Therefore, a combined strategy is required in disease prevention, surveillance, and control to reduce the double burden of diseases. Notably, malaria is a preventable disease, applying mosquito repellent to exposed skin and using mosquito netting over beds could effectively prevent malaria [38]. Also, research suggested that the onset of some NCDs is closely related to lifestyle habits [7]. Primary prevention-the adoption of healthy lifestyles is critical to slow down the onset and exacerbation of diseases, such as cardiovascular disease and type II diabetes mellitus [39, 40]. Secondary prevention-early detection, diagnosis and treatment on the other hand, can improve symptoms, reduce mortality and disability, and prevent the recurrence of diseases, such as type I diabetes mellitus, chronic kidney disease, Alzheimer’s disease and other dementias [41, 42].

Additionally, the increases of mortality rates for many NCDs are tended to be in younger population. There is growing evidence that improving children and adolescents’ attitudes and awareness is a healthy way to live and prevent disease as they grow up [43]. Health promotion is one out of many pubic health actions for both CDs and NCDs prevention [44, 45]. Activities surrounding the social determinants of health or “upstream” activities that modify the environment could make them either less susceptible to communicable disease, or more oriented towards in a healthy lifestyle are much more effective in the long run to reduce the double burden of diseases [46]. Health systems strengthening constitutes an important strategy that apply to the NCDs prevention and control. For instance, as ones of six building blocks modularized by WHO, flexibly accessible financing and health workforce serve as key input components for maintaining a resilient health system that is prepared for prevention and control of NCDs as well as communicable diseases; while a health information or surveillance system allows the policy maker to grasp the situation and make adjustment timely [47]. Promoting primary health care, with significance attached to people-centered service delivery, is most essential and cost-effective approaches towards universal health coverage and tackling with the rising and double burden of NCDs and communicable diseases [48].

Notwithstanding the world is currently facing many challenges in communicable diseases and NCDs prevention and control, it is fortunate that WHO estimates that a large number of deaths can be avoided through preventive interventions [7, 49]. Essential public health technologies, including prevention, screening, early detection, and disease management of risk behavioral factors in primary healthcare, play an unprecedented role in making healthcare more accessible and reducing the disease burden of NCDs, especially in LMICs [50]. Recent research has found that with the rapid increase in the burden of NCDs in sub-Saharan Africa over the past few decades, many governments have issued policies related to NCDs and launched relative programs [51]. However, many policies and programs have not been implemented due to the fragile health system, insufficient health workforce, and inadequate staff capacity [52]. There are also a large regional and subnational differences in the program implementation [53]. Public health technology development and health system strengthening should be considered at the same time in order to promote disease management for non-communicable and communicable diseases. Enhanced efforts, collaboration and communication among regional and national policymakers, international organizations and other stakeholders are required to ensure future policy and implementation improvements.

This study had several limitations. The GBD 2019 public dataset were based on secondary data from existing registriers [22]. Some estimates of cross-sectional data might be reflected by wide uncertainly intervals. Primary data collection should to be strengthened in places with sparse and absent data to improve the research accuracy [54]. Meanwhile, this study reviewed mortality trends of NCDs in the top ten malaria burden countries in Africa. Further analyses are required to investigate different high mortality communicable diseases and NCDs across the African countries to obtain more comprehensive results.

## Conclusion

In summary, we found that countries with the highest burden of malaria also face a large burden of NCDs. Urgent attention needs to be paid to the structural factors behind the convergence of non-communicable and communicable diseases, and a combined strategy is required in disease prevention, surveillance, and control. Jointly exploring the double burden of diseases in LMICs through multilateral partnership and cooperation is warranted, and national governments and international organizations should be aligned and supported by the global initiative.

## Electronic supplementary material

Below is the link to the electronic supplementary material.

Supplementary Material 1

Supplementary Material 2

## Data Availability

All data used in this study can be freely accessed at the GBD 2019 portal (http://ghdx.healthdata.org/gbd-2019).

## List of abbreviations

NCDs: Non-communicable diseases
HIV: Human immunodeficiency virus
GBD: Global Burden of Disease
TB: Tuberculosis
ASMR: Age-standardised mortality rate
DALY: Disability-adjusted life-years
YLL: Years of life lost
YLD: Years lost due to a disability
EAPC: Estimated annual percentage change
WHO: World Health Organization’s
UN: United Nations
LMICs: Low- and middle-income countries
IHME: Institute for Health Metrics and Evaluation
ASR: Age-standardized rates
CI: Confidence interval
SPSS: Statistical Package for social science
