# Supplementary material for: Temporal trends in the burden of non-communicable diseases in countries with the highest malaria burden, 1990–2019: Evaluating the double burden of non-communicable and communicable diseases in epidemiological transition
Source: Global Health. 2022 Oct 23;18:90. doi: 10.1186/s12992-022-00882-w (PMC9589679; doi:10.1186/s12992-022-00882-w)
Supplement: Supplementary file 1 — Supplementary Material 1 [file 12992_2022_882_MOESM1_ESM.docx]

**Appendix table 1.** Contribution of the top five NCDs with the highest mortality rate to the total mortality rate of each country, by sex, in 2019 (%)

| Location | Gender | Alzheimer's disease and other dementias | Chronic kidney disease | Chronic obstructive pulmonary disease | Cirrhosis and other chronic liver disease | Diabetes Mellitus | Hypertensive heart disease | Ischemic heart disease | Stroke | Total |
| --- | --- | --- | --- | --- | --- | --- | --- | --- | --- | --- |
| Benin | Both | -- | 1.70 | -- | 2.09 | 1.65 | -- | 4.76 | 5.17 | 15.37 |
|  | Female | -- | 1.71 | -- | 1.71 | 1.94 | -- | 5.14 | 5.83 | 16.34 |
|  | Male | -- | 1.69 | -- | 2.40 | 1.41 | -- | 4.45 | 4.61 | 14.56 |
| Burkina Faso | Both | -- | 1.40 | -- | 1.48 | 1.47 | -- | 4.69 | 3.67 | 12.72 |
|  | Female | -- | 1.53 | -- | 1.35 | 1.35 | -- | 4.48 | 3.82 | 12.53 |
|  | Male | -- | 1.29 | -- | 1.59 | 1.58 | -- | 4.88 | 3.53 | 12.87 |
| Cameroon | Both | -- | 2.31 | -- | 1.99 | 2.50 | -- | 5.12 | 5.23 | 17.16 |
|  | Female | -- | 2.40 | -- | 1.48 | 2.74 | -- | 4.99 | 5.43 | 17.05 |
|  | Male | -- | 2.23 | -- | 2.42 | 2.30 | -- | 5.23 | 5.06 | 17.25 |
| Cote d'Ivoire | Both | -- | 1.88 | -- | 2.16 | 1.93 | -- | 5.29 | 4.72 | 15.98 |
|  | Female | -- | 1.75 | -- | 1.51 | 2.16 | -- | 5.08 | 4.85 | 15.35 |
|  | Male | -- | 1.98 | -- | 2.64 | 1.75 | -- | 5.44 | 4.63 | 16.45 |
| Liberia | Both | -- | 2.17 | -- | 3.21 | 2.13 | -- | 6.35 | 5.39 | 19.25 |
|  | Female | -- | 2.26 | -- | 2.43 | 2.50 | -- | 6.67 | 5.90 | 19.76 |
|  | Male | -- | 2.08 | -- | 3.90 | 1.80 | -- | 6.07 | 4.95 | 18.79 |
| Mozambique | Both | 0.58 | -- | -- | -- | 1.78 | 1.54 | 3.37 | 6.03 | 13.30 |
|  | Female | 0.91 | -- | -- | -- | 1.49 | 2.13 | 3.02 | 5.78 | 13.33 |
|  | Male | 0.31 | -- | -- | -- | 1.80 | 1.06 | 3.66 | 6.24 | 13.28 |
| Niger | Both | -- | -- | 0.88 | 1.64 | 1.04 | -- | 3.33 | 3.53 | 10.42 |
|  | Female | -- | -- | 0.80 | 1.34 | 1.28 | -- | 3.52 | 3.97 | 10.91 |
|  | Male | -- | -- | 0.96 | 1.90 | 0.83 | -- | 3.17 | 3.13 | 9.99 |
| Nigeria | Both | 0.76 | -- | -- | 2.53 | 1.54 | -- | 4.37 | 3.98 | 13.18 |
|  | Female | 0.92 | -- | -- | 1.84 | 1.50 | -- | 4.53 | 4.39 | 13.18 |
|  | Male | 0.31 | -- | -- | 2.01 | 1.06 | -- | 4.22 | 3.63 | 13.18 |
| Sierra Leone | Both | -- | 1.48 | -- | 1.70 | 1.56 | -- | 5.59 | 5.13 | 15.46 |
|  | Female | -- | 1.48 | -- | 1.37 | 1.96 | -- | 6.11 | 5.84 | 16.77 |
|  | Male | -- | 1.47 | -- | 1.99 | 1.21 | -- | 5.13 | 4.51 | 14.31 |
| Togo | Both | -- | 1.88 | -- | 2.38 | 1.95 | -- | 6.93 | 5.98 | 19.11 |
|  | Female | -- | 1.90 | -- | 1.96 | 2.33 | -- | 7.25 | 6.73 | 20.16 |
|  | Male | -- | 1.85 | -- | 2.73 | 1.63 | -- | 6.65 | 5.33 | 18.20 |
| Global | Both | 2.87 | 2.52 | 5.80 | 2.60 | 2.74 | 2.05 | 16.17 | 11.59 | 46.35 |
|  | Female | 4.12 | 2.65 | 5.41 | 1.95 | 3.08 | 2.56 | 16.13 | 12.46 | 48.37 |
|  | Male | -- | 1.85 | -- | 2.73 | 1.63 | -- | 16.19 | 10.86 | 44.66 |

Note: mark “--” is used if the death rate of corresponding disease is not ranked top 5 in the countries.

**Appendix table 2.** Estimated annual percentage change of ASMR for eight major NCDs in ten selected countries, 1990-2009.

| Country | Disease | | | | | | | |
| --- | --- | --- | --- | --- | --- | --- | --- | --- |
|  | Ischemic heart disease | | | Stroke | | | | |
|  | EAPC (%) | 95% CI (%) | P | EAPC (%) | 95% CI (%) | | | P |
| Benin | -0.3015 | (-0.3590, -0.2441) | <0.001 | -0.6638 | (-0.7397, -0.5879) | | | <0.001 |
| Burkina Faso | 0.9061 | (0.7055, 1.1071) | <0.001 | 0.4066 | (0.2957, 0.5176) | | | <0.001 |
| Cameroon | 0.4608 | (0.2604, 0.6616) | <0.001 | -0.1671 | (-0.3986, 0.0649) | | | =0.151 |
| Cote d'Ivoire | -0.6063 | (-0.7273, -0.4850) | <0.001 | -0.6821 | (-0.8285, -0.5354) | | | <0.001 |
| Liberia | -0.3692 | (-0.5136, -0.2246) | <0.001 | -0.7952 | (-0.8938, -0.6967) | | | <0.001 |
| Mozambique | 1.2337 | (1.0350, 1.4329) | <0.001 | 0.6883 | (0.5092, 0.8675) | | | <0.001 |
| Niger | -0.2707 | (-0.3846, -0.1567) | <0.001 | -0.4938 | (-0.5608, -0.4267) | | | <0.001 |
| Nigeria | -0.8012 | (-0.9259, -0.6762) | <0.001 | -1.5302 | (-1.6892, -1.3708) | | | <0.001 |
| Sierra Leone | -0.0012 | (-0.1441, 0.1419) | =0.986 | -0.0308 | (-0.1735, 0.1123) | | | =0.663 |
| Togo | -0.1013 | (-0.1619, -0.0408) | =0.002 | -0.3288 | (-0.4138, -0.2437) | | | <0.001 |
| Global | -1.3569 | (-1.3944, -1.3193) | <0.001 | -1.7354 | (-1.8653, -1.6054) | | | <0.001 |
| Country | Diabetes mellitus | | | Diabetes mellitus (Type I) | | | | |
|  | EAPC (%) | 95%CI (%) | P | EAPC (%) | 95%CI (%) | | | P |
| Benin | 0.8560 | (0.628, 1.085) | <0.001 | -0.9195 | （-1.1144，-0.7242） | | | <0.001 |
| Burkina Faso | -0.0608 | (-0.165, 0.044) | =0.243 | -0.6569 | （-1.1863，-0.1248） | | | =0.017 |
| Cameroon | 1.0983 | (0.794, 1.403) | <0.001 | -0.5626 | (-0.6819, -0.4433) | | | <0.001 |
| Cote d'Ivoire | 0.3262 | (-0.010, 0.663) | =0.057 | -0.9098 | (-1.0356, -0.7839) | | | <0.001 |
| Liberia | 0.5369 | (0.394, 0.680) | <0.001 | -0.9678 | (-1.4320, -0.5012) | | | <0.001 |
| Mozambique | 1.0653 | (0.918, 1.213) | <0.001 | 0.8631 | (0.6328, 1.0939) | | | <0.001 |
| Niger | 0.6567 | (0.509, 0.804) | <0.001 | -0.8097 | (-1.0609, -0.5579) | | | <0.001 |
| Nigeria | 0.3200 | (0.204, 0.436) | <0.001 | -0.9999 | (-1.3379, -0.6606) | | | <0.001 |
| Sierra Leone | 1.1976 | (0.967, 1.429) | <0.001 | 0.0000 | (-0.251, 0.2034) | | | =0.830 |
| Togo | 0.7010 | (0.488, 0.915) | <0.001 | -0.5982 | (-0.7968, -0.4988) | | | <0.001 |
| Global | 0.1893 | (0.090, 0.289) | =0.001 | -1.0101 | (-1.1025, -0.9177) | | | <0.001 |
| Country | Diabetes mellitus (Type II) | | | Chronic kidney disease | | | | |
|  | EAPC (%) | 95%CI (%) | P | EAPC (%) | | 95%CI (%) | P | |
| Benin | 1.0050 | (0.7025, 1.2072) | <0.001 | -0.1526 | | (-0.2377, -0.0675) | =0.001 | |
| Burkina Faso | 0.0000 | (-0.1000, 0.1001) | =0.633 | 0.0321 | | (-0.0738, 0.1381) | =0.540 | |
| Cameroon | 1.2072 | (0.8529, 1.4997) | <0.001 | -0.2898 | | (-0.3664, -0.2131) | <0.001 | |
| Cote d'Ivoire | 0.4008 | (0.0328, 0.7522) | =0.034 | -0.6080 | | (-0.7323, -0.4837) | <0.001 | |
| Liberia | 0.6018 | (0.5013, 0.8032) | <0.001 | -0.5848 | | (-0.8656, -0.3031) | <0.001 | |
| Mozambique | 1.1061 | (0.904, 1.2072) | <0.001 | 0.4963 | | (0.3602, 0.6327) | <0.001 | |
| Niger | -0.7968 | (-1.0609, -0.5579) | <0.001 | -0.6635 | | (-0.7311, -0.5959) | <0.001 | |
| Nigeria | 0.4008 | (0.3005, 0.5013) | <0.001 | -0.2954 | | (-0.3596, -0.2311) | <0.001 | |
| Sierra Leone | 1.2720 | (1.0050, 1.5113) | <0.001 | -0.2782 | | (-0.3637, -0.1926) | <0.001 | |
| Togo | 0.8032 | (0.5464, 1.0197) | <0.001 | -0.4308 | | (-0.4713, -0.3903) | <0.001 | |
| Global | 0.2641 | (0.1606, 0.3679) | <0.001 | 0.5505 | | (0.4544, 0.6466) | <0.001 | |
| Country | Chronic liver diseases | | | Alzheimer’s disease and other dementias | | | | |
|  | EAPC (%) | 95%CI (%) | P | EAPC (%) | 95%CI (%) | | | P |
| Benin | -1.4994 | (-1.668, -1.331) | <0.001 | 0.0063 | (-0.053, 0.065) | | | =0.828 |
| Burkina Faso | -2.3266 | (-2.636, -2.016) | <0.001 | -0.1202 | (-0.175, -0.065) | | | <0.001 |
| Cameroon | -1.7486 | (-1.977, -1.520) | <0.001 | 0.0751 | (0.055, 0.095) | | | <0.001 |
| Cote d'Ivoire | -1.5699 | (-1.769, -1.371) | <0.001 | 0.0806 | (0.041, 0.121) | | | <0.001 |
| Liberia | -1.4317 | (-1.571, -1.292) | <0.001 | 0.1507 | (0.083, 0.219) | | | <0.001 |
| Mozambique | -0.7032 | (-0.950, -0.456) | <0.001 | 0.5788 | (0.544, 0.613) | | | <0.001 |
| Niger | -1.2230 | (-1.376, -1.070) | <0.001 | 0.3925 | (0.352, 0.433) | | | <0.001 |
| Nigeria | -0.6378 | (-0.828, -0.447) | <0.001 | 0.8468 | (0.711, 0.983) | | | <0.001 |
| Sierra Leone | -2.2184 | (-2.466, -1.970) | <0.001 | 0.3453 | (0.309, 0.382) | | | <0.001 |
| Togo | -1.6740 | (-1.887, -1.461) | <0.001 | 0.0849 | (0.070, 0.100) | | | <0.001 |
| Global | -1.1215 | (-1.215, -1.028) | <0.001 | 0.1258 | (0.098, 0.154) | | | <0.001 |
| Country | Hypertensive heart disease | | | | Chronic obstructive pulmonary disease | | | |
|  | EAPC (%) | 95%CI (%) | P | EAPC (%) | 95%CI (%) | | | P |
| Benin | 0.8344 | (0.450, 1.220) | <0.001 | -1.0474 | (-1.209, -0.886) | | | <0.001 |
| Burkina Faso | 1.8933 | (1.551, 2.237) | <0.001 | -0.6145 | (-0.721, -0.508) | | | <0.001 |
| Cameroon | 0.0132 | (-0.376, 0.404) | =0.945 | -1.1755 | (-1.291, -1.060) | | | <0.001 |
| Cote d'Ivoire | 0.8736 | (0.432, 1.318) | <0.001 | -1.1945 | (-1.318, -1.071) | | | <0.001 |
| Liberia | 0.7961 | (0.510, 1.083) | <0.001 | -0.6116 | (-0.786, -0.437) | | | <0.001 |
| Mozambique | 0.1041 | (-0.073, 0.281) | =0.239 | -0.1493 | (-0.293, -0.005) | | | =0.043 |
| Niger | 0.0788 | (-0.208, 0.367) | =0.579 | -0.3867 | (-0.567, -0.206) | | | <0.001 |
| Nigeria | -0.9632 | (-1.315, -0.610) | <0.001 | -0.2254 | (-0.396, -0.055) | | | =0.012 |
| Sierra Leone | 1.2941 | (0.885, 1.705) | <0.001 | -0.5082 | (-0.710, -0.306) | | | <0.001 |
| Togo | 0.8632 | (0.519, 1.209) | <0.001 | -0.7452 | (-0.910, -0.580) | | | <0.001 |
| Global | -0.7432 | (-0.913, -0.573) | <0.001 | -2.1272 | (-2.235, -2.019) | | | <0.001 |

Note: EAPC, Estimated Annual Percentage Change; ASMR, age-standardized mortality rate.
