# Supplementary material for: Temporal trends in the burden of non-communicable diseases in countries with the highest malaria burden, 1990–2019: Evaluating the double burden of non-communicable and communicable diseases in epidemiological transition
Source: Global Health. 2022 Oct 23;18:90. doi: 10.1186/s12992-022-00882-w (PMC9589679; doi:10.1186/s12992-022-00882-w)
Supplement: Supplementary file 2 — Supplementary Material 2 [file 12992_2022_882_MOESM2_ESM.docx]

**Appendix figure 1**. Type I diabetes mellitus ASMR trends in the ten countries and globally, 1990-2019

**Appendix figure 2.** Type II diabetes mellitus ASMR trends in the ten countries and globally, 1990-2019.


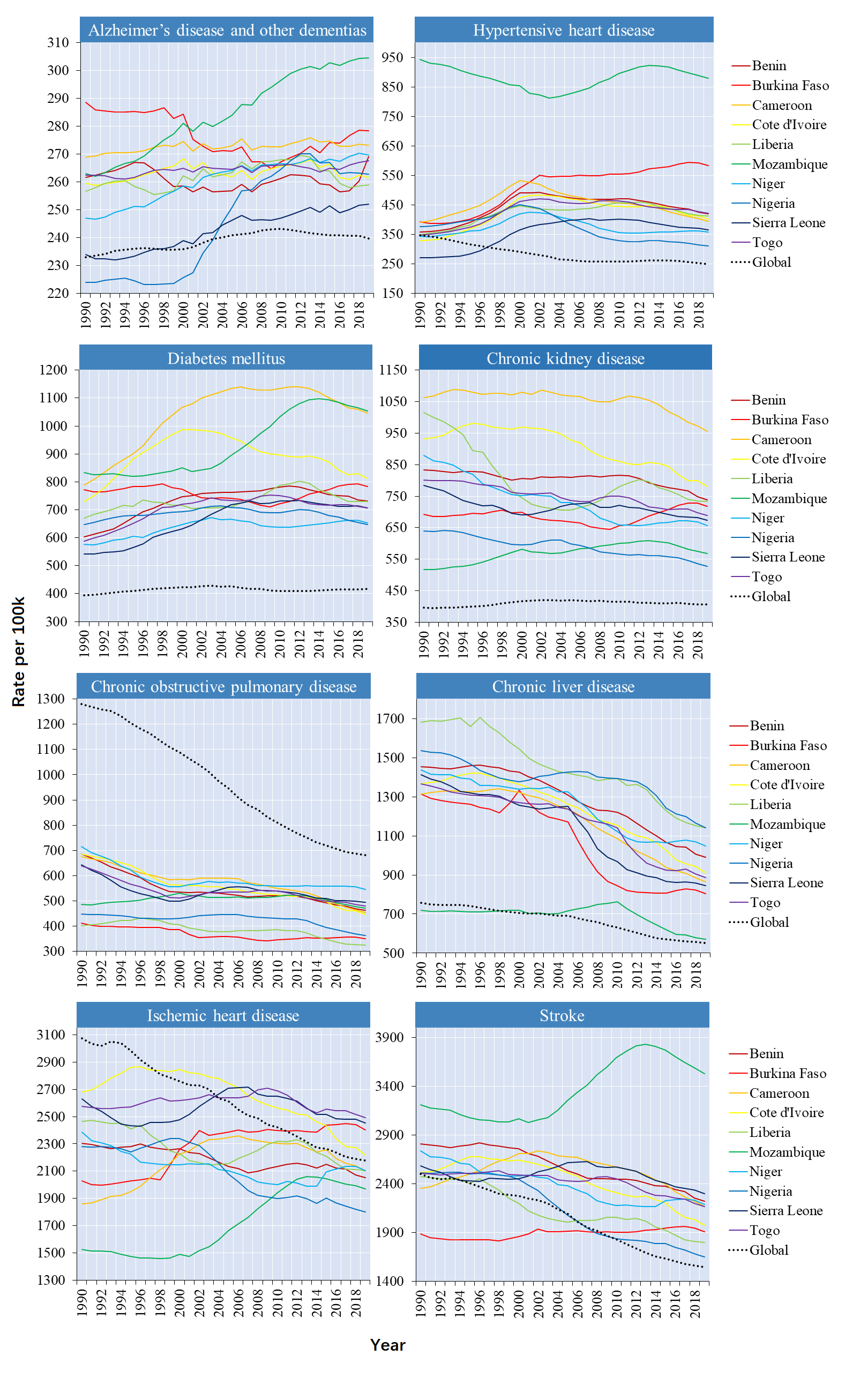
**Appendix figure 3**. Trends of age-standardized rates for YLLs of the eight selected NCDs in the ten countries per 100 000 populations, 1990-2019


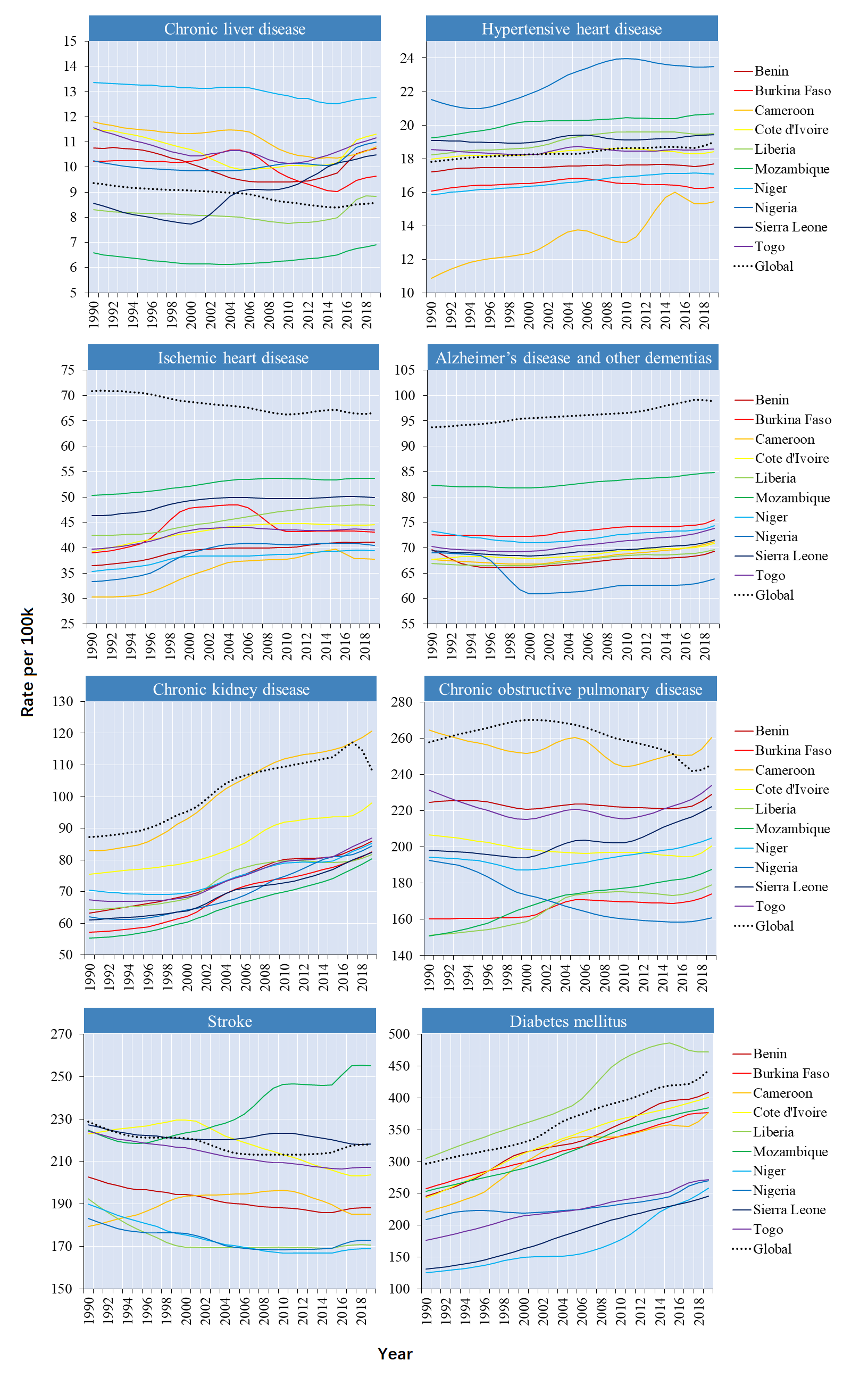
**Appendix figure 4**. Trends of age-standardized rates for YLDs of the eight selected NCDs in the ten countries per 100 000 populations, 1990-2019.
